# Brief Group Cognitive‐Behavioral Therapy for Non‐Underweight Eating Disorders: Feasibility and Preliminary Effectiveness

**DOI:** 10.1002/eat.24572

**Published:** 2025-10-13

**Authors:** Jill L. L. Bluff, Ellie K. Daly, Isabelle R. Bird, Hazel Bryce, Sally Brook, Jessica Beard

**Affiliations:** ^1^ South Yorkshire Eating Disorders Association Sheffield UK; ^2^ Department of Psychology University of Sheffield Sheffield UK

**Keywords:** early change, group CBT‐T, group cognitive‐behavioral therapy, nonunderweight eating disorders, service outcomes

## Abstract

**Objective:**

Individually delivered 10‐session cognitive‐behavioral therapy for nonunderweight eating disorders (CBT‐T) has demonstrated comparable levels of effectiveness to longer CBT‐ED. Group CBT‐T has demonstrated feasibility and potential effectiveness in a pilot study. This study assessed the effectiveness and feasibility of group CBT‐T in a larger sample of adults, and evaluated the predictive value of early change on treatment outcomes.

**Method:**

The data analysis was pre‐registered and received ethical clearance, and sample size analysis requirements were met. Using intention to treat analyses (ITT), generalized linear mixed models were used to examine change in eating disorder psychopathology, depression, anxiety, and objective binge eating. Recovery, reliable improvement, and clinically significant change were also examined. Early response as a predictor of treatment outcome was assessed with a paired samples *t*‐test and Pearson's product‐moment correlation.

**Results:**

Fifty‐nine patients started group CBT‐T and were entered into the ITT analyses. Twenty‐two (37.3%) patients did not complete therapy. Eating disorder psychopathology, depression, anxiety, and objective binge eating significantly reduced from pre‐ to post‐therapy (sustained at 3‐month follow‐up) with medium to very large effect sizes. Of the treatment completers (*n* = 37, 62.7%), over 70% recovered on the EDE‐Q, and over half showed reliable improvement and clinically significant change. Patients who showed early change in EDE‐Q scores by session 4 had significantly greater mean changes in EDE‐Q scores from session 1 to session 10.

**Discussion:**

The present study shows that group CBT‐T can be effective in reducing eating disorder psychopathology and objective binge eating frequency, and improves mood in a transdiagnostic sample of patients with non‐underweight eating disorders. Group CBT‐T has the potential to increase accessibility to evidence‐based treatment for nonunderweight eating disorders.

**Summary**

- This study evaluated the preliminary effectiveness and feasibility of group‐based CBT‐T for non‐underweight eating disorders in a routine mild‐to‐moderate outpatient setting.
- Feasibility, as measured by attrition, was comparable to individually delivered CBT‐T, and patients showed clinically and statistically significant reductions in eating disorder psychopathology, mood, and objective binge eating frequency over the course of CBT‐T.
- These findings further support the effectiveness of group‐based CBT‐T, but larger controlled studies are needed.

## 1 Introduction

Cognitive‐behavioral therapy for eating disorders (CBT‐ED) is the first line of treatment recommended by the National Institute for Health and Care Excellence [NICE] (2017) for non‐underweight eating disorders in adults. Its efficacy has been demonstrated in controlled research trials (e.g., Fairburn et al. 2009, 2015), and it has shown strong levels of effectiveness in routine clinical settings (e.g., Byrne et al. 2011; Knott et al. 2015; Signorini et al. 2018).

However, CBT‐ED protocols initially required 20 sessions (e.g., Fairburn 2008), which is considerably longer than treatments recommended for other disorders (e.g., 8–10 sessions for depression and anxiety; Clark 2018). Furthermore, patients have reported receiving substantially more sessions than the evidence‐based recommended number, for example, 45 sessions rather than 20 in treating Bulimia Nervosa (BN) (Cowdrey and Waller 2015). Thus, while CBT‐ED is effective, it is expensive to deliver and has implications in terms of waiting times for treatment. This is further exacerbated when services need to prioritize the treatment of underweight eating disorders to manage risk. This means waiting times for the treatment of patients who are not underweight (e.g., Binge‐eating disorder (BED), BN, atypical cases) are often excessively long.

In 2017, NICE recommended evaluating briefer treatments for eating disorders. In keeping with this recommendation, Waller et al. (2019) developed CBT‐T, a 10‐session CBT‐ED for nonunderweight eating disorders. Rather than aiming to treat specific eating disorder diagnoses, CBT‐T targets eating disorder psychopathology (eating, weight, and shape concerns and related behaviors). Individually delivered CBT‐T has been tested across a number of studies (case series, cohort studies, randomized controlled trials) and samples (BED, BN, atypical cases), demonstrating medium to very large effect sizes for eating disorder psychopathology, clinical impairment, and mood (Keegan et al. 2022; Paphiti and Newman 2023). Crucially, CBT‐T has demonstrated comparable levels of effectiveness to versions of CBT‐ED that are twice as long (Tatham et al. 2020). CBT‐T has also been adopted more widely within the First Episode Rapid Early Intervention for Eating Disorders (FREED) initiative, with nearly a quarter of patients within the FREED network receiving CBT‐T, which performed comparably to longer CBT‐ED (Allen et al. 2025).

Brief therapies such as CBT‐T have the potential to reduce waiting times, increase access to therapy, and reduce costs to services. However, delivering therapy in a group format has the potential to further such benefits. Group CBT‐ED has demonstrated similar levels of effectiveness compared to individual CBT‐ED (e.g., Ricca et al. 2010; Wade et al. 2017). However, group CBT‐ED is still typically relatively long. One study has investigated the feasibility, compliance, and acceptability of brief online group CBT‐T (Moore and Waller 2023). A third of patients invited took part in the group, demonstrating limited acceptance. However, compliance and feasibility of the treatment were strong, with all eight treatment starters completing the therapy. While this study hinted at the potential clinical effectiveness of group CBT‐T, the sample was too small to reach any firm conclusion. It is therefore important to determine the effectiveness and feasibility of group CBT‐T with a larger sample.

It is also important to determine whether the impact of early change in eating disorders (Chang et al. 2021; Keegan and Wade 2024; Vall and Wade 2015) is replicated in a group context. In CBT‐T, patients are collaboratively discharged from the therapy if they have not demonstrated early progress by session 4 (Waller et al. 2019). This review point is similar to the ‘taking stock’ phase in CBT‐E (Fairburn 2008). Patients are made aware of the review point at the start of treatment. This approach aims to motivate patients to make early change in order to progress further in therapy. Where there is a lack of progress at session 4, the patient is given the opportunity to make necessary changes before session 5, and it is reiterated that early change is the only reliable predictor of treatment outcome. Collaboratively ending therapy at this point ensures patients do not feel they have “failed” at therapy, or had their time wasted, as the evidence suggests that continuing with therapy in the absence of early change results in poorer therapeutic outcomes for these patients. Discussions are held with the patient to discuss whether now is the right time for therapy, or whether a different therapeutic approach or service is more appropriate. Ending treatment at this point also enables services and clinicians to allocate resources more efficiently (Waller et al. 2019). But does this pattern hold true in a group context, where early change might be limited by the less individualized approach?

Hence, the first aim of the current study was to assess the feasibility and effectiveness of group CBT‐T for nonunderweight eating disorder patients amongst a larger sample. The second was to determine whether early change remains a robust predictor of outcomes when delivered in a group format. It was hypothesized that: (a) group CBT‐T would lead to significant reductions in eating disorder psychopathology, depression, and anxiety, comparable to the effect sizes seen in individually delivered CBT‐T; (b) early change in eating disorder psychopathology by session 4 would be associated with better outcomes at session 10.

## 2 Method

### 2.1 Design

An uncontrolled open‐label design was used to evaluate the effectiveness of group CBT‐T for non‐underweight eating disorders for adults in a routine outpatient setting. Outcomes were assessed at baseline (session 1), session 4, at the end of therapy (session 10), and at 3‐month follow‐up. Data were collected between January 2024 and July 2025.

### 2.2 Ethical Considerations

As this study conducted a retrospective analysis on anonymous routinely collected service data, full ethical approval was not required (National Health Service Research Authority 2017). A self‐declaration ethics application was approved by Sheffield University Research Ethics Committee (065840). All patients consented to the use of their anonymous data for service evaluation. This study was pre‐registered (https://osf.io/wt5ah), and deviations from that protocol are noted below.

### 2.3 Patients

Patients (*N* = 59) either self‐referred or were referred by a clinician (e.g., family physician, therapist) to South Yorkshire Eating Disorders Association (SYEDA) for eating disorder treatment. SYEDA is a mild‐to‐moderate outpatient service. Following referral, all patients were assessed to ensure they did not meet the exclusion criteria for treatment at SYEDA. These criteria include severe vomiting or laxative use (> 5 episodes a week), Body Mass Index (BMI = weight kg/height m^2^) < 17.5 kg/m^2^, excessively abusing alcohol or substances, attempting to complete suicide within the last 6 months, having had bariatric surgery within the last 6 weeks (or still unable to eat a minimum of three solid meals per day), currently using weight loss injections (unless prescribed for the management of diabetes), and active suicidality.

At assessment, the following treatment offers were discussed: CBT‐T, Occupational Therapy, and Counseling. Following treatment selection, patients were placed onto the appropriate waiting list. All patients on the waiting list for CBT‐T were invited to take part in the group (unless patients specified at assessment that they did not wish to have group therapy). Patients could elect to join group CBT‐T with minimal waiting time, or wait the standard longer time for individual CBT‐T. Inclusion criteria for group CBT‐T included any eating disorder diagnosis provided patients had a BMI ≥ 18.5, age ≥ 17 years, being willing to pause any weight loss throughout the duration of therapy, and not receiving any other mental health treatment throughout the duration of the therapy. Exclusion criteria included those listed above.

### 2.4 Treatment

#### 2.4.1 Group CBT‐T Protocol

Previously, CBT‐T was adapted for group delivery, retaining the structure and content of the protocol, but with adaptations in delivery (Moore and Waller 2023). However, that group protocol required each patient to receive a weekly 10‐min one‐to‐one session in addition to the group session, making it less feasible for use with larger groups. In 2024, JLLB developed group CBT‐T version 2, which places greater emphasis on equipping patients with the tools and knowledge to act as their own therapist, reducing time spent individually with group participants. Each weekly session lasted up to 90 min, and patients reviewed their own progress and identified personal areas for behavioral change.

Patients were invited to attend either an in‐person group (two clinicians attended in person) or an online group via Zoom or Microsoft Teams (two clinicians attended virtually). This mix in treatment delivery was to allow for patients across South Yorkshire to still attend group CBT‐T if they were unable to travel to the main SYEDA clinic. Patients were unable to swap between the online and in‐person groups once they had started treatment.

It was agreed that no more than 10 patients would be assigned to a group to ensure group sizes were not too big. There was no predetermined minimal number of patients to start a group, though it was desirable to have at least seven to allow for drop‐out and still be efficient to run with two clinicians. Groups continued if there were fewer patients than this to honor the offer of starting group CBT‐T.

Patients who missed one or two sessions were provided with catch‐up recordings of the session, enabling them to complete the required homework and be ready for the following week's session. Patients who missed more than two sessions were discharged from the group and added back to the waiting list to access either a later group or individual CBT‐T when able to commit to the weekly sessions. However, for patients who felt that the time wasn't right to continue with any therapy, it was mutually agreed that they would be discharged from the service.

The group CBT‐T materials are freely available at https://cbt‐t.sites.sheffield.ac.uk/group‐cbt‐t. The main adaptations to Moore and Waller's (2023) protocol are presented in Table S1.

#### 2.4.2 Therapists

Each group was delivered by two clinicians who were trained in CBT‐T, or by a CBT‐T clinician and an Occupational Therapist familiar with the CBT‐T protocol. Six therapists in total were involved. Each received weekly or fortnightly supervision by a clinician experienced in treating eating disorders and who had attended training in CBT‐T. Therapists were guided by the protocol (Waller et al. 2019) and slides developed by JLLB.

### 2.5 Measures

Patients completed three measures at session 1, 4, 10, and at two follow‐up sessions (1‐ and 3‐months post therapy). For this study, data collected at session 1, 4, 10, and the 3‐month follow‐up session were analyzed.

The *Eating Disorder Examination‐Questionnaire* (EDE‐Q 6.0; Fairburn and Beglin 2008) was used to assess eating disorder psychopathology. The EDE‐Q includes four subscales (restraint, eating concerns, weight concerns, shape concerns), and the average of the subscales yields a global score. Each subscale and the global score range from 0 to 6, with higher scores indicating greater eating disorder psychopathology. The EDE‐Q subscales have demonstrated acceptable to high internal consistency (Berg et al. 2012).

The Personal Health Questionnaire (PHQ‐9; Kroenke et al. 2001) and Generalized Anxiety Disorder scale (GAD‐7; Spitzer et al. 2006) were used to measure depression and anxiety. The total PHQ‐9 score ranges from 0 to 27, and the total GAD‐7 score ranges from 0 to 21, with higher scores indicating greater severity. The PHQ‐9 and GAD‐7 have demonstrated excellent internal consistency (Kroenke et al. 2001; Spitzer et al. 2006).

Weekly objective binge eating (OBE), vomiting, and laxative frequencies were recorded at each session, as were weekly predicted and actual weight changes. Online patients emailed this information to the group facilitators ahead of each session. Face‐to‐face patients shared this information with the group facilitators when they were weighed individually each week. Session 1 covered the difference between objective and subjective binge eating. Patients were also reminded of the differences between objective and subjective binge eating when asked to provide their OBE frequency each week.

### 2.6 Data Analysis

Data were analyzed using IBM SPSS v29. Descriptive statistics were used to analyze demographic characteristics (age, gender, ethnicity, eating disorder presentation), and reason for attrition. Paired samples t‐tests were used to examine changes in BMI. Feasibility of the intervention was assessed using the rate of attrition. Group CBT‐T was deemed to have good feasibility if attrition rates were comparable to those found in individual CBT‐T (23%–50%; Paphiti and Newman 2023).

#### 2.6.1 Eating Disorder Psychopathology, Mood, and OBE Frequency

To avoid problems of correlations between variables over time, Generalized Linear Mixed Models (GLMM) were used instead of repeated measures ANOVAs to assess changes in eating disorder psychopathology, mood, and frequency of OBE. GLMM is not impacted by time‐point interdependence of dependent variables and maximizes the use of all available data. Multiple imputation was not used as GLMM is robust with respect to missing data (Twisk et al. 2013). Cohen's *d* was used to determine effect sizes, based on the means and standard deviations of the differences between the time points for each outcome.

Sample size analysis (G*Power v.3.1.9.2) was carried out to assess how many patients would be necessary to detect a medium effect size (*f* = 0.25). Assuming *p* = 0.05 and Power = 80%, a total sample size of *N* = 24 was necessary for a one‐way repeated measures design over four time points. The current study was thus adequately powered to detect a medium effect size.

#### 2.6.2 Recovery, Reliable Improvement, and Clinically Significant Change

Percentage of patients achieving recovery, reliable improvement, and clinically significant change on the GAD‐7, PHQ‐9, and EDE‐Q was also assessed. Recovery was defined as moving from “caseness” (being above a clinical threshold) to “no caseness” (being below a clinical threshold). Caseness was defined as scores ≥ 8 for the GAD‐7, and ≥ 10 for the PHQ‐9 (Porter et al. 2024). Caseness for the EDE‐Q global was defined as scores ≥ 2.77 (Mond et al. 2004). Reliable improvement for the GAD‐7 and PHQ‐9 was based on scores outlined by NHS Talking Therapies: a decrease of ≥ 4 on the GAD‐7 and ≥ 6 on the PHQ‐9 (Porter et al. 2024). Reliable improvement for the EDE‐Q was ≥ 1.38 (Moore et al., n.d.). Clinically significant change was defined as achieving both recovery and reliable change. To enable comparisons with other CBT‐ED studies, “good outcome” on the EDE‐Q Global was also assessed. Consistent with other studies, good outcome was defined as having a post‐treatment EDE‐Q Global score < 2.77.

#### 2.6.3 Early Change as a Predictor of Outcome

To assess whether early change in EDE‐Q Global scores by session 4 predicted outcomes at session 10, two methods of identifying early responders were used. Method 1: a Pearson product–moment correlation was used to assess whether continuous change scores on the EDE‐Q Global from session 1 to session 4 were associated with change scores from session 1 to session 10. Method 2: based on Wade et al. (2025), patients were considered early responders if their EDE‐Q Global scores reduced by ≥ 1.13 points by session 4. Independent samples *t*‐tests were used to assess whether early responders showed greater change in EDE‐Q Global scores from session 1 to session 10 compared to nonearly responders.

### 2.7 Deviations From the Pre‐Registration

The pre‐registration outlines two aims to be assessed using two datasets. Aim 1 was to assess the pre‐post effectiveness of CBT‐T using paired samples *t*‐tests on data collected by SYEDA and any other participating services. Aim 2 was to assess the predictive value of early change by session 4 using GLMM on data collected only by SYEDA (because other services were not collecting data at session 4). Due to constraints in other services, only data collected by SYEDA were used for this study; thus, only one dataset was used to address both aims. GLMM (rather than *t*‐tests) was therefore used to assess the pre‐post effectiveness of group CBT‐T, but with the addition of session 4 and follow‐up data. Pearson's correlations and independent samples *t*‐tests were used to assess the predictive value of early change.

Although the current study was adequately powered to detect a medium effect size, due to the open‐labeled uncontrolled design, this study is now presented as a feasibility and preliminary effectiveness study. Measures of feasibility were not predetermined in the pre‐registration.

## 3 Results

Table 1 shows the pre‐treatment demographic and clinical characteristics of the 59 patients who started group CBT‐T (across eight online groups and two face‐to‐face groups; mean *n* = 5.9 patients per group). Forty‐four (74.6%) patients were in online groups, and 15 (25.4%) were in face‐to‐face groups. Most clients were White British females, with a mean age of 34.6 years. Most had binge‐eating disorder. It is notable that patients from a non‐White background had higher baseline EDE‐Q Global scores than patients from a White background, though the sample is too limited to draw any firm conclusions. The mean baseline EDE‐Q Global scores were similar across each diagnosis.

**TABLE 1:** Pre‐treatment patient demographic information.

Demographic/characteristic	*N* (%)	Mean (SD) baseline EDE‐Q Global
Age (years)	M = 34.6, SD = 11.7, range 17–68 years	
Gender		
Male	9 (15.3)	2.84 (1.3)
Female	49 (83.1)	3.76 (1.0)^a^
Other	1 (1.7)	3.53 (0.0)
Ethnicity		
Any White background	52 (88.1)	3.50 (1.1)^b^
Any Black background	3 (5.1)	4.43 (0.55)
Any Asian background	2 (3.4)	4.31 (1.1)
Other	2 (3.4)	4.28 (0.7)
Diagnosis		
BED	36 (61.0)	3.50 (1.0)^c^
OSFED	9 (15.3)	3.61 (1.3)
BN	9 (8.5)	3.90 (1.4)
UFED	5 (8.5)	3.82 (1.0)
BMI	M = 36.1, SD = 11.0, range 19.3–65.3	

*Note*: Due to missing EDE‐Q Global data at session 1.

Abbreviations: BED, Binge‐Eating Disorder; BMI, body mass index; BN, Bulimia Nervosa; M, mean; OSFED, other specified feeding and eating disorder; SD, standard deviation; UFED, unspecified feeding and eating disorder.

^a^ *n* = 47.

^b^ *n* = 50.

^c^ *n* = 34.

### 3.1 Feasibility (Patient Attrition)

Table 2 shows the attrition rates for patients who started group CBT‐T. The overall percentage of patients who dropped out of treatment was 37.3%, which is comparable to the rates reported in a systematic review of individual CBT‐T (23%–50%; Paphiti and Newman 2023), indicating good feasibility. The most common reason for dropout was “ended by mutual agreement.” This included reasons such as no longer being able to commit to the weekly sessions or starting another therapy at another service (e.g., for depression, etc.). Patients who “did not attend” (DNA) were those who stopped attending therapy without giving a reason and were thus discharged from the service. Only 5.1% of patients who started group CBT‐T decided to transfer to individually delivered CBT‐T, and 5.1% were transferred to counseling. No patients were collaboratively discharged at session 4 as a result of a lack of early change. Patients who remained in treatment from session 1 to session 10 but did not attend the follow‐up session(s) were not considered as having dropped out.

**TABLE 2:** Reason for patient attrition (total *n* = 22, 37.3%).

Reason for attrition	*N* (%)	Mean baseline EDE‐Q Global
Ended by mutual agreement	11 (18.6)	4.00 (1.0)
Transferred to alternative treatment	3 (5.1)	4.15 (0.8)
Did not attend (DNA)	5 (8.5)	4.18 (1.3)^a^
Transferred to one‐to‐one CBT‐T	3 (5.1)	3.69 (1.1)
Total	22 (37.3)	4.01 (1.0)^b^

*Note*: Due to missing EDEQ‐Global data at session 1.

^a^ *n* = 4.

^b^ *n* = 21.

Binary logistic regression found no significant predictors of attrition (EDE‐Q subscales, EDE‐Q Global, PHQ‐9, GAD‐7, age, BMI) of drop out (*Χ* ^2^ = 11.193 (df = 9), *p* = 0.263).

### 3.2 Treatment Outcomes

#### 3.2.1 Intention to Treat Analyses

Table 3 shows the number of patients with data at each time point, and the means and standard deviations for each outcome measure at each time point.

**TABLE 3:** Mean scores and standard deviations at each time point.

Time	*N*	Mean	SD
EDE‐Q Global			
S1	57	3.61	1.10
S4	48	2.19	1.24
S10	35	1.56	1.22
3‐month FU	27	1.24	1.01
PHQ‐9			
S1	58	13.50	6.05
S4	50	8.74	5.44
S10	34	6.35	5.14
3‐month FU	23	6.13	5.61
GAD‐7			
S1	58	10.07	5.33
S4	50	6.88	5.14
S10	34	4.97	4.45
3‐month FU	23	5.17	5.34
Objective binge‐eating frequency			
S1	51	2.78	3.67
S4	44	0.93	1.40
S10	31	0.16	0.45
3‐month FU	20	0.40	0.82

**FIGURE 1:**
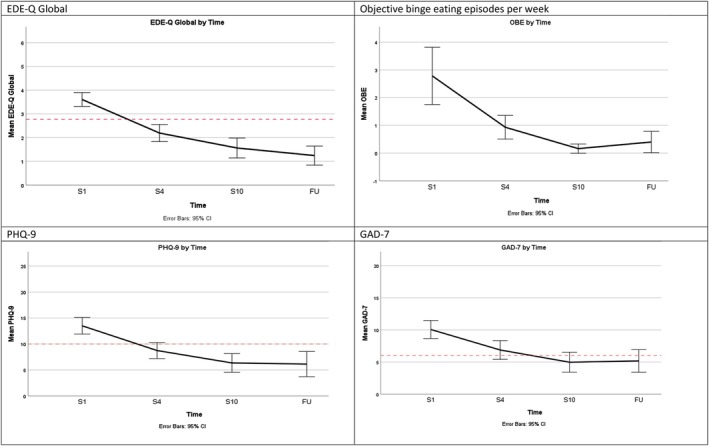
GLMM results—mean scores across group CBT‐T and at 3‐month follow up. The red dotted line indicates clinical threshold.

Results from the GLMM analyses are shown in Table 4. Scores on each outcome measure (EDE‐Q, PHQ‐9, GAD‐7) significantly decreased from beginning to session 4, to the end of group CBT‐T (session 10), and at the 3‐month follow‐up. The GLMM results for each of the EDE‐Q subscales can be found in Table S2. The effect sizes (Cohen's *d*) ranged from medium to very large effect sizes, with particularly large effects for the core outcome variable of EDE‐Q Global scores. OBE also significantly decreased over time. Due to the limited number of patients vomiting or using laxatives at the beginning of treatment, data on these behaviors are not presented. No patients were using these behaviors at session 10 or at the 3‐month follow up. Figure 1 shows the scores (estimated marginal means and 95% confidence intervals) for each measure. Figure 1 shows that the mean EDE‐Q Global, PHQ‐9, and GAD‐7 scores moved from above to below the clinical threshold for each measure from session 1 to session 4, and remained below threshold at session 10 and the 3‐month follow up. The scores for each of EDE‐Q subscales can be found in Figure S1.

**TABLE 4:** GLMM results for each outcome: change from start of therapy to S4, S10, and 3‐month follow up.

Fixed effect of time	B	SE	95% CI	*p*	*d*
EDE‐Q Global					
S4	−1.414	0.227	−1.862, −0.967	< 0.001	0.86
S10	−2.044	0.248	−2.534, −1.554	< 0.001	1.25
3‐month FU	−2.363	0.270	−2.897, −1.830	< 0.001	1.59
PHQ‐9					
S4	−4.760	1.086	−6.905, −2.615	< 0.001	0.59
S10	−7.147	1.216	−9.548, −4.746	< 0.001	0.90
3‐month FU	−7.370	1.387	−10.109, −4.630	< 0.001	0.89
GAD‐7					
S4	−3.189	0.954	−5.073, −1.305	< 0.001	0.43
S10	−5.098	1.068	−7.207, −2.990	< 0.001	0.73
3‐month FU	−4.895	1.218	−7.300, −2.490	< 0.001	0.73
OBE					
S4	−1.852	0.481	−2.804, −0.901	< 0.001	0.47
S10	−2.623	0.533	−3.676, −1.570	< 0.001	0.71
3‐month FU	−2.384	0.617	−3.604, −1.165	< 0.001	0.63

#### 3.2.2 Completer Analyses

Thirty‐seven patients (62.7%) completed 10 sessions of group CBT‐T. Of these 37 patients, 29 (78.4%) attended the 1‐month follow up, and 23 (62.2%) attended the 3‐month follow up.

##### 3.2.2.1 Body Mass Index

For the 37 treatment completers, no significant difference in BMI was found from session 1 (M = 35.2, SD = 11.3) to session 10 (M = 35.3, SD = 11.3), *p* = 0.555. Nor did it change significantly from session 1 to the 3‐month follow up (*n* = 17 due to missing data; M = 30.2, SD = 12.6), *p* = 0.296.

##### 3.2.2.2 Objective Binge Eating

Table 3 shows the mean number of objective binges at each time point. Forty‐one patients (69.5%) were bingeing at session 1. Of the 50 patients still in treatment at session 4, 17 (34%) were still bingeing. Of the 37 treatment completers, four (10.8%) were still bingeing at session 10. Of the 23 patients who attended the 3‐month follow‐up, five (21.7%) were still bingeing.

##### 3.2.2.3 Recovery, Reliable Improvement, and Clinically Significant Change by the End of Therapy

Table 5 shows the percentage of patients achieving *recovery* (moving from above to below clinical threshold), reliable improvement (reducing ≥ 4 points on the GAD‐7, ≥ 6 on the PHQ‐9, and ≥ 1.38 on the EDE‐Q Global), and clinically significant change (showing both recovery and reliable improvement) at session 10.

**TABLE 5:** Percentage of patients achieving recovery, reliable improvement, and clinically significant change at session 10.

	EDE‐Q Global *N* (%)	PHQ‐9 *N* (%)	GAD‐7 *N* (%)
Recovery (crosses threshold)^a^			
Recovered	18 (72.0)	16 (64.0)	10 (52.6)
Not recovered	7 (28.8)	8 (32.0)	8 (42.1)
Missing S10 data	0	1 (4.0)	1 (5.3)
Reliable improvement^b^			
Reliably improved	22 (59.5)	22 (59.5)	15 (40.5)
Not reliably improved	12 (32.4)	12 (32.4)	19 (51.4)
Missing S10 data	3 (8.1)	3 (8.1)	3 (8.1)
Clinically significant change (achieves recovery and reliable improvement)^a^			
Clinically significant change	15 (60.0)	16 (64.0)	8 (42.1)
No clinically significant change	10 (40.0)	8 (33.3)	10 (52.7)
Missing S10 data	0	1 (4.0)	1 (5.3)

^a^ Percentage of patients who started treatment above threshold.

^b^ Percentage of all completers.

Of the 37 patients who completed group CBT‐T, 25 (67.6%) started treatment above the clinical threshold on the EDE‐Q Global and on the PHQ‐9, and 19 (51.4%) on the GAD‐7. Twenty‐eight completers (75.7%) had achieved “good outcome” at session 10 (EDE‐Q Global scores < 2.77; two patients had missing session 10 data). Recovery and reliable improvement rates were very strong for eating disorder psychopathology and mood, particularly depression. Over half of the patients who started group CBT‐T above clinical threshold on the EDE‐Q Global achieved both recovery and reliable improvement (clinically significant change) by session 10. No patients showed reliable deterioration on any measure.

### 3.3 Predictive Value of Early Change

Using completer analyses (and excluding cases with missing data), two operationalisations of early change were used to assess whether change in the first four weeks of therapy predicted outcomes at session 10.

#### 3.3.1 Continuous Measure of Early Change

A Pearson product–moment correlation found a strong positive correlation between change in EDE‐Q Global scores from session 1 to session 4 and change in scores from session 1 to session 10, which was statistically significant (*r* = 0.772, *n* = 34, *p* < 0.001). The change in EDE‐Q Global scores from session 1 to session 4 was not significantly correlated with changes from session 4 to session 10 (*r* = −0.151, *n* = 34, *p* = 0.394). Thus, early change in EDE‐Q scores appears to account for the majority of overall change across therapy.

#### 3.3.2 Dichotomous Measure of Early Change

Based on Wade et al. (2025), patients were considered early responders if their EDE‐Q Global scores reduced by ≥ 1.13 points by session 4. Using this definition of early change, 21 (56.8%) patients were categorized as early responders (very similar to the 58% reported by Wade et al. 2025). An independent samples t‐test found that early responders had statistically significantly greater reductions in their EDE‐Q Global scores from session 1 to session 10 (M_change_ = 2.33, SD = 0.93) compared to non‐early responders (M_change_ = 1.13, SD = 0.75), *t*(32) = 4.011, *p* = < 0.001. Again, this finding supports the need to focus on early change to maximize the impact of CBT‐T in such cases.

## 4 Discussion

This study examined the feasibility and preliminary effectiveness of group CBT‐T for non‐underweight eating disorders and assessed whether early change was a predictor of treatment outcome. As hypothesized, patients showed clinically and statistically significant reductions in eating disorder psychopathology, mood, and objective binge eating frequency by session 4, which was sustained throughout therapy and at 3‐month follow‐up. Attrition rates were comparable to those reported for individual CBT‐T, indicating good feasibility. Recovery and reliable improvement rates were strong, particularly for eating disorder psychopathology and depression. Nearly 60% of patients were identified as early responders, which was associated with greater mean changes in EDE‐Q Global scores from beginning to end of treatment.

The results of this study extend those reported by Moore and Waller (2023), suggesting that group CBT‐T is a feasible alternative to individually delivered CBT‐ED. This study addressed some of the limitations outlined in the pilot study. Notably, the current study was adequately powered to detect a medium effect size and assessed the effectiveness of the intervention using an intention‐to‐treat approach. However, the current study also used an open‐labeled uncontrolled design; thus, this study still indicates preliminary evidence of effectiveness. Patients in the current study were made aware of the group CBT‐T treatment offer at assessment, which might have improved uptake, though the number of patients who declined group CBT‐T was not recorded. Finally, the current study included diagnoses other than BED and BN (i.e., OSFED and UFED), increasing generalizability to other non‐underweight eating disorders. However, due to the exclusion criteria of the service (e.g., purging > 5× per week), we can be more firm about the conclusions for BED. Similarly, due to small subgroup sizes, it was not possible to conclude whether outcomes varied by diagnosis. Patients with atypical anorexia nervosa (AAN) have demonstrated poorer outcomes in individually delivered CBT‐T (Keegan and Wade 2024), but due to having very few patients with AAN in the current sample, it was not possible to evaluate whether the same applies for group therapy. Further studies with larger samples and fewer exclusion criteria are needed to assess whether group CBT‐T outcomes vary by diagnosis and whether adaptations to the group protocol could be made to enhance outcomes for these individuals.

The findings of this study are comparable to those of individually delivered CBT‐T, suggesting the effectiveness of the intervention is not compromised when delivered in a group setting. For example, the medium to very large effect sizes found in this study are similar to those reported in systematic reviews and meta‐analyses of individual CBT‐T (Keegan et al. 2022; Paphiti and Newman 2023). Similarly, rates of recovery, reliable improvement, and ‘good outcome’ reported in the current study reflect those found in individual CBT‐T. For example, 75.7% of treatment completers achieved good outcome at post‐treatment, compared to a pooled 65% reported in a meta‐analysis of individual CBT‐T (Keegan et al. 2022). The preliminary effectiveness of group CBT‐T is also comparable to that of group enhanced CBT (CBT‐E) for non‐underweight eating disorders (Wade et al. 2017). However, group CBT‐E was conducted over 18 two‐hour sessions. Consistent with research into individually delivered CBT‐T, the current study suggests brief group CBT‐T (delivered in 10 90‐min sessions) performs comparably to group CBT‐ED but in almost half the time.

Considering early change, 56.85% of the sample was classed as early responders (similar to 58% identified in an individual CBT‐T sample; Wade et al. 2025). Consistent with individually delivered CBT‐T (Keegan and Wade 2024; Wade et al. 2025) and eating disorder treatment more generally (Chang et al. 2021), early change was associated with better outcomes when delivered in a group format. No patients were discharged from group CBT‐T at session 4, despite 43.2% of patients not showing early change on the EDE‐Q Global score (Wade et al. 2025), because early change in group CBT‐T was assessed by changes made to eating, rather than simple changes in EDE‐Q Global scores. Keegan and Wade (2024) found that early change was still a predictor of CBT‐T outcome even when patients who were collaboratively discharged at session 4 were excluded from their completer analyses. This warrants further consideration in order to explore the most appropriate index of early change in group CBT‐T.

## 5 Limitations and Future Research

The current findings should be considered in the context of their limitations. First, due to service limitations it was not possible to record the patient flow from assessment to starting group CBT‐T. However, similar to individually delivered CBT‐T (e.g., Paphiti and Newman 2023; Pellizzer et al. 2019; Waller et al. 2019), attrition rates in the current study were relatively low, indicating good acceptance once in treatment. Future studies should further explore the uptake of group CBT‐T. Similarly, this study did not examine patient perspectives on the intervention. Future studies should examine patient experiences of group CBT‐T, to assess whether it reflects those of individual CBT‐T (Hoskins et al. 2019). Such understanding has the potential to increase rates of uptake of group CBT‐T in the future.

Although the current study was adequately powered to detect a medium effect size, it is limited in terms of its generalizability to more diverse populations. For example, the current sample was predominantly White adult women with Binge‐Eating Disorder. It is important to examine whether group CBT‐T outcomes are robust across more diverse populations. Similarly, this study only used data from one mild‐to‐moderate outpatient service. Future research should include data from multiple sites to ensure that the results are generalizable to other outpatient services.

The data in this study were collected retrospectively, and patients were not randomly assigned to either individual or group CBT‐T. Instead, patients self‐selected group CBT‐T from other treatment options available during their assessment, remaining on the waiting list if they were happy to wait longer for individual CBT‐T. This could have biased the sample in a positive direction towards group CBT‐T. However, the outcomes still suggest that those who opt for group CBT‐T generally do well. Nonetheless, group CBT‐T should be evaluated using a randomized design, and should consider a follow‐up period longer than 3 months.

A particular strength of this study is that patients were not receiving any other treatment. Therefore, changes to mood can likely be attributed to the effects of group CBT‐T, despite not being a specific treatment target. However, it is notable that a significant number of patients were lost to follow‐up. It was not feasible to assess the reasons for this, which is something future research should consider, as those with better outcomes might have been more likely to attend follow‐ups. Finally, it is worth noting that the nature of the treating clinicians suggests that a range of professionals could successfully deliver the intervention with appropriate training and supervision.

## 6 Conclusion

This study provides further evidence for the feasibility of CBT‐T and its effectiveness, delivered both online and in person. Group CBT‐T has minimal reliance on time spent individually with patients. This has implications in terms of availability and access to evidence‐based treatment, potentially reducing waiting times and improving service cost‐effectiveness, without compromising treatment effectiveness. Group CBT‐T also has the potential to be implemented as part of early access initiatives.

## Author Contributions

**Jill L. L. Bluff:** conceptualization, investigation, writing – original draft, methodology, writing – review and editing, resources. **Ellie K. Daly:** conceptualization, investigation, writing – original draft, methodology, writing – review and editing, resources. **Isabelle R. Bird:** conceptualization, investigation, writing – original draft, methodology, writing – review and editing. **Hazel Bryce:** conceptualization, writing – original draft, writing – review and editing, investigation, methodology. **Sally Brook:** investigation, conceptualization, writing – review and editing. **Jessica Beard:** conceptualization, writing – original draft, writing – review and editing, formal analysis, project administration, methodology, investigation, supervision.

## Conflicts of Interest

The authors declare no conflicts of interest.

## Supporting information


**Data S1:** eat24572‐sup‐0001‐Sipinfo.docx.

## Data Availability

The data that support the findings of this study are available from the corresponding author upon reasonable request.
