# Supplementary material for: Brief Group Cognitive‐Behavioral Therapy for Non‐Underweight Eating Disorders: Feasibility and Preliminary Effectiveness
Source: Int J Eat Disord. 2025 Oct 13;59(1):146–55. doi: 10.1002/eat.24572 (PMC12773681; doi:10.1002/eat.24572)
Supplement: Supplementary file 1 — Data S1: eat24572‐sup‐0001‐Sipinfo.docx. [file EAT-59-146-s001.docx]

**Supplementary materials**

**Supplementary Table 1.** Adaptations made to group CBT-T (Moore & Waller, 2023) for this study.

| **Aspect of CBT-T group** | **Adaptations made** |
| --- | --- |
| Length and structure of sessions | Sessions were restructured into two parts: Part one covered largely the same material as the original group CBT-T protocol (Moore & Waller, 2023). Part two contained additional materials and was labelled ‘weekly skills session’. This part was designed to provide patients with the skills and knowledge to become their own therapists.  Sessions typically lasted 90 minutes. However, there was an opportunity for patients to speak privately with a therapist after each session if needed. |
| Presentation of psychoeducation | A new document was created labelled ‘pre-course resource’. This included all the relevant psychoeducation and templates required for each session. This was emailed to group members one week before session 1.  New weekly handouts were created including information required for each of the skills sessions. The relevant weekly handout was emailed to patients the day before each session (or provided on the day for face-to-face patients). |
| Tracking of weight changes and eating disorder behaviour frequency | Previously, group CBT-T patients had weekly 10 minute online one-to-one sessions with a clinician before the start of the session. This time was used to check patients’ self-reported estimated and actual weight change, review binge/purge frequencies, and examine food diaries.  In the newly developed protocol, patients did not meet with clinicians individually prior to weekly group sessions. Instead, the rationale for weighing and how this is done in CBT-T was covered in the first skills session.  Patients attending online group CBT-T were given responsibility for recording their own actual and predicted weights and binge/purge frequencies in their pre-course resource booklet. They were asked to email that information to clinicians prior to each weekly session. Patients attending face-to-face CBT-T were weighed individually by clinicians during the session coffee break. Clinicians also asked them for their weekly binge/purge frequencies. Clinicians recorded this information and also invited patients to record the information in their own pre-course resource booklet.  Calculation and reporting of group mean weight change and binge/purge frequencies over the week was included in the group sessions as before. In future groups, the group average weight and OBE/purge frequencies will no longer be shared due to the risk of outliers skewing the data. Instead, patients will be asked to plot their own individual graphs using the data they record in their pre-course resource booklet.  Patients received a copy of changes to their individual outcome measures at session 4 and session 10 in order to provide feedback and encouragement that their behavioural changes were reflected in measurable improvements in eating disorder psychopathology. |
| Monitoring of eating behaviours (food diaries) | Patients were provided with the flexible CBT-T food plan to start following from session 1, and were asked to record their own daily intake on food diaries. In the updated protocol, patients were trained in how to analyse food diaries in the second weekly skills session (rather than individually with a clinician ahead of each session).  Patients were trained to check for gaps longer than 3-4 hours between meals and snacks, to ensure they had eaten 3 meals and 2-3 snacks every day, and to ensure each meal and snack included complex carbohydrates. It was highlighted that alongside the food diary, patients could use their weekly weight as an indicator of whether they are eating enough to maintain their body weight, and reduce the risk of bingeing (though weekly weight fluctuations were also discussed). In every subsequent session, patients had time to analyse their food diary on their own before feeding back as a group about anything significant they had noticed (e.g., triggers to bingeing). As a safeguard, patients emailed their most recent food diary to the group facilitators just prior to session 4. The facilitators could then ensure that patients were eating in accordance with the CBT-T food plan, and could continue with CBT-T. Individual feedback on food diaries were provided here. |
| Slide content | The first two CBT-T groups included in the present study used the same slides as those developed by Moore and Waller (2023), though was delivered using the structure outlined above (no individual sessions). However, for subsequent groups, slides were amended to include additional material. Sensitivity analysis found no difference in GLMM outcomes when removing patients from these two groups.  All materials and slides are available at <https://cbt-t.sites.sheffield.ac.uk/group-cbt-t> |

**Supplementary Table 2.** GLMM results for EDE-Q subscales: change from start of therapy to S4, S10, and 3-month follow up.

| Fixed effect of time | B | SE | 95% CI | *p* | *d* |
| --- | --- | --- | --- | --- | --- |
| **EDE-Q Restriction** | | | | |  |
| S4 | -1.537 | .284 | -2.097, -.976 | < .001 | .71 |
| S10 | -1.739 | .311 | -2.353, -1.124 | < .001 | .80 |
| 3 month FU | -1.820 | .358 | -2.527, -1.113 | < .001 | .88 |
| **EDE-Q Eating Concerns** | | | | |  |
| S4 | -1.732 | .267 | -2.259, -1.206 | < .001 | .83 |
| S10 | -2.335 | .293 | -2.912, -1.757 | < .001 | 1.21 |
| 3 month FU | -2.501 | .336 | -3.166, -1.837 | < .001 | 1.35 |
| **EDE-Q Weight Concern** | | | | |  |
| S4 | -1.227 | .265 | -1.749, -.704 | < .001 | .63 |
| S10 | -2.018 | .290 | -2.591, -1.445 | < .001 | 1.10 |
| 3 month FU | -2.272 | .334 | -2.931, -1.613 | < .001 | 1.34 |
| **EDE-Q Shape Concern** | | | | |  |
| S4 | -1.212 | .279 | -1.763, -.662 | < .001 | .61 |
| S10 | -2.098 | .306 | -2.701, -1.495 | < .001 | 1.08 |
| 3 month FU | -2.531 | .351 | -3.225, -1.837 | < .001 | 1.33 |

**Supplementary Figure 1.** GLMM results – mean EDE-Q subscale scores across group CBT-T and at 3-month follow up.

| EDE-Q Restraint | EDE-Q Eating Concern |
| --- | --- |
| 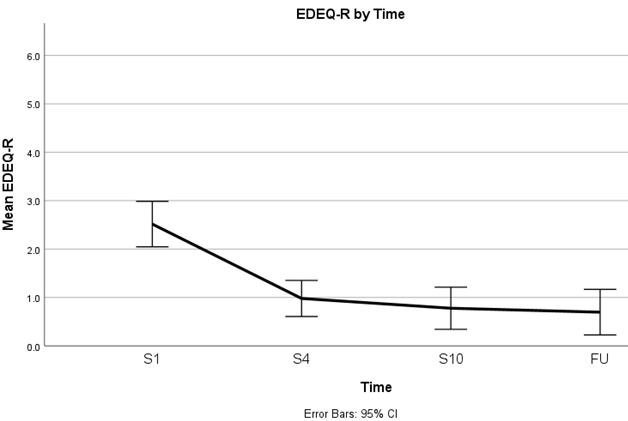 | 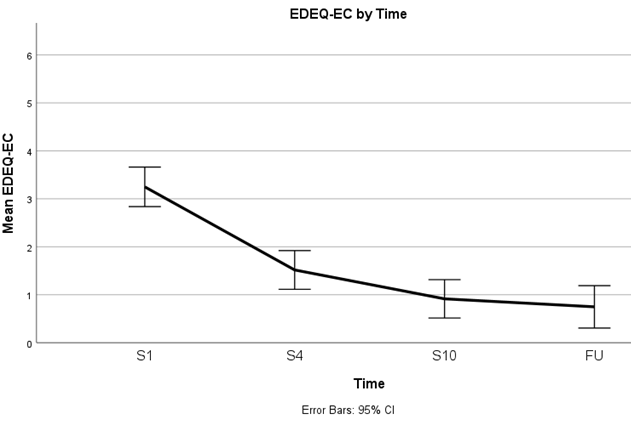 |
| EDE-Q Shape Concern | EDE-Q Weight Concern |
| 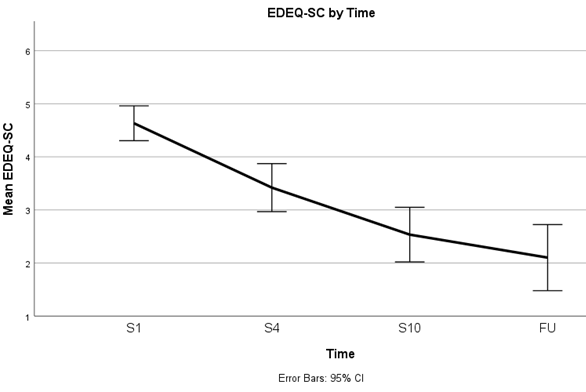 | 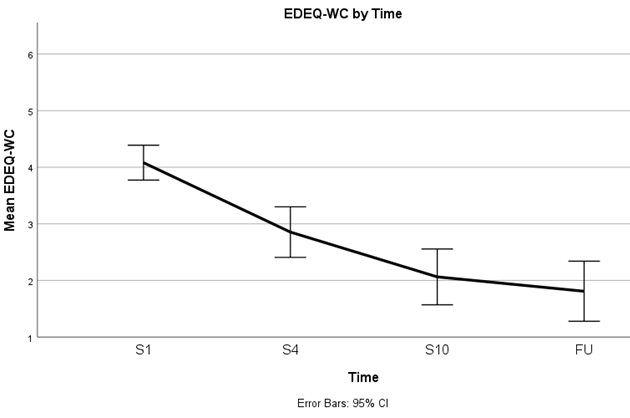 |
